# Meta-analysis and indirect treatment comparison of modified FOLFIRINOX and gemcitabine plus nab-paclitaxel as first-line chemotherapy in advanced pancreatic cancer

**DOI:** 10.1186/s12885-021-08605-x

**Published:** 2021-07-23

**Authors:** Jiayuan Chen, Qingling Hua, Haihong Wang, Dejun Zhang, Lei Zhao, Dandan Yu, Guoliang Pi, Tao Zhang, Zhenyu Lin

**Affiliations:** 1 Cancer Center, Union Hospital, Tongji Medical College, Huazhong University of Science and Technology, Wuhan, 430022 China; 2 Department of Radiation Oncology, Hubei Cancer Hospital, Tongji Medical College, Huazhong University of Science and Technology, Wuhan, 430079 China

**Keywords:** Pancreatic cancer, Modified FOLFIRINOX, Gemcitabine, Nab-paclitaxel, Network meta-analysis, Systematic review

## Abstract

**Background:**

Modified FOLFIRINOX and gemcitabine plus nab-paclitaxel (GEM-NAB) have been recommended as first-line therapies for advanced pancreatic cancer (PC). Due to the lack of evidence to directly compare them, we conducted this network meta-analysis to indirectly compare the effectiveness and toxicity of modified FOLFIRINOX and GEM-NAB.

**Methods:**

The eligible retrospective studies on treatments related to modified FOLFIRINOX and GEM-NAB up to 4 April 2020 were searched and assessed. We used the frequentist model to analyze the survival and toxicity data between different treatments. Pooled analysis for overall survival (OS), progression-free survival (PFS), objective response rate (ORR) and events of toxicity were analyzed in this study.

**Results:**

Twenty-two studies were involved in this network meta-analysis. The comparisons on OS and PFS showed that modified FOLFIRINOX and GEM-NAB had similar treatment efficacy (**OS**: 1.13; 95% CI: 0.78–1.63; **PFS**: HR: 1.19; 95% CI: 0.85–1.67). GEM-NAB was more effective than modified FOLFIRINOX based on the result of ORR (RR: 1.43; 95% CI: 1.04–1.96). Moreover, our analysis showed a similar toxicity profile between modified FOLFIRINOX and GEM-NAB.

**Conclusions:**

The current evidence showed that modified FOLFIRINOX and GEM-NAB were similar in survival and toxicity. Many factors should be considered for in the formulation of optimal treatment, and our meta-analysis could provide some guidance to treatment selection in the first-line setting for advanced PC.

**Supplementary Information:**

The online version contains supplementary material available at 10.1186/s12885-021-08605-x.

## Background

Among all cancer-related deaths, pancreatic cancer (PC) ranked fourth in both men and women in the United States. Despite the significant improvements in therapeutic strategies, the prognosis of PC was still poor with a 5-year survival rate less than 7% as most patients were diagnosed with advanced-stage disease. There will be an estimated 57,600 new cases and 47,050 deaths with PC in 2020 [1]. As curative surgery could only be performed in less than 20% of cases, chemotherapy was commonly used in patients with locally advanced or metastatic PC. Gemcitabine (GEM) were found to have more clinical benefits than 5-fluorouracil (5-FU) on treating advanced PC and became one of the main chemotherapeutic drugs. Chemotherapies based on gemcitabine or fluoropyrimidine are conventional in the treatments for advanced PC [2, 3]. Different anti-tumor agents were combined with gemcitabine or fluoropyrimidine in numerous clinical trials to improve treatment efficacy. And gemcitabine combined with nab-paclitaxel prolonged overall survival (OS) for around two months than gemcitabine monotherapy [4].

Among all regimen combinations, FOLFIRINOX (folinic acid, 5-FU, irinotecan and oxaliplatin) and GEM-NAB (gemcitabine plus nab-paclitaxel) have currently shown great benefits as first-line therapies for advanced PC. In general, GEM-NAB is more tolerable and preferred by older patients with a higher Eastern Cooperative Oncology Group (ECOG) score, while FOLFIRINOX is often used in younger patients [5, 6]. Due to drug toxicities, dosage modifications have been made in FOLFIRINOX, and modified FOLFIRINOX have been recommended by several institutions [7, 8]. In particular, evidence to directly assess the benefits or adverse effects of modified FOLFIRINOX or GEM-NAB as first-line treatments was lacking. Thus, we collected data from observational retrospective studies, and performed a systematic review and network meta-analysis in this article. We aimed to compare the effectiveness and toxicity of modified FOLFIRINOX and GEM-NAB indirectly in the first-line setting.

## Methods

### Literature search and article selection

We conducted a systematic search to find available papers in literature. The databases including PubMed, Embase, Cochrane and Web of Science were independently searched by two investigators from inception to April 2020. We utilized the following keywords for this search: pancreatic cancer, gemcitabine, nab-paclitaxel, and modified FOLFIRINOX. The search strategy was as follows: (((‘folinic acid’/exp. AND fluorouracil/exp. AND irinotecan/exp. AND oxaliplatin/exp. AND ‘drug combination’/exp) OR (Folfirinox):ab,ti) OR (gemcitabine/exp. AND ‘Albumin-Bound Paclitaxel’/exp. ‘drug combination’/exp)) AND (‘pancreas cancer’/de OR ‘pancreas tumor’/de OR ‘pancreas adenoma’/de OR ‘pancreas adenocarcinoma’/de OR ‘pancreas carcinoma’/de OR ‘pancreas islet cell carcinoma’/de OR (pancrea* NEAR/3 (cancer* OR neoplas* OR tumo* OR adenocarcinom* OR carcinom* OR adenom*)):ab,ti). The Additional file 2 listed detailed search strategy.

**Fig. 1:**
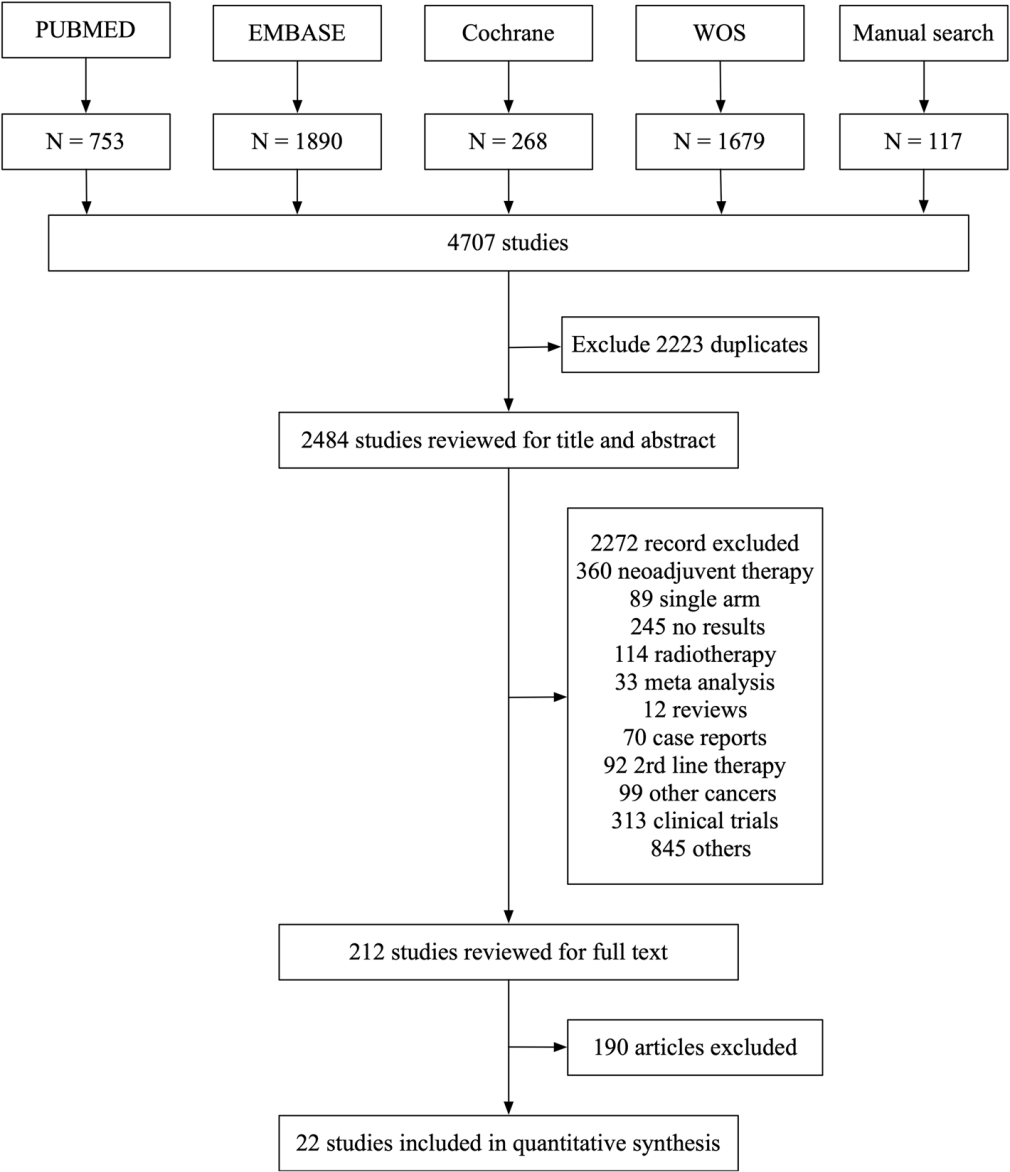
Flow chart for study search (PRISMA diagram)

Our search was supplemented by a manual search for relevant studies. The detailed flow diagram of inclusion and exclusion process was presented in Fig. 1. We included studies on human species written in English. We selected retrospective studies to compare the benefits and adverse effects/safety of modified FOLFIRINOX versus GEM-NAB of patients with locally advanced or metastatic PC. Standard regimen of FOLFIRINOX included oxaliplatin 85 mg/m^2^, leucovorin 400 mg/m^2^, irinotecan 180 mg/m^2^, 5-FU bolus 400 mg/m^2^ and 5-FU 2400 mg/m^2^. The dose of one regimen at least was reduced in modified FOLFIRINOX with/without the removal of 5-FU bolus [9]. We excluded studies if they were duplicates, reviews, case reports, meta-analysis or having no association with our research.

### Data extraction

General information was extracted from the selected studies by two investigators. Any disagreements regarding the extraction of data were resolved by discussions among several investigators. The following fields were abstracted: the name of article, the name of first author, country, year of publication, number of patients, characteristics of patients (age and sex), beginning and ending time, median followed-up time, treatment efficacy data as well as safety data.

OS was chosen as the primary endpoint. Progression-free survival (PFS), objective response rate (ORR), and grade ≥ 3 adverse events (according to National Cancer Institute Common Terminology Criteria for Adverse Events version 4.0) were selected as the secondary endpoints. The hazard ratios (HRs) with 95% confidence intervals (CIs) of PFS and OS were extracted from the selected publications. The total events of complete response (CR), partial response (PR), stable disease (SD), and progressive disease (PD) were also extracted to evaluate ORR to chemotherapy. The grade ≥ 3 adverse events included neutropenia, febrile neutropenia, thrombocytopenia, anemia, anorexia, fatigue, nausea, and diarrhea.

### Statistical analysis

Frequentist model was used in this analysis [10, 11]. Differences in efficacy between modified FOLFIRINOX and GEM-NAB were assessed by HRs with 95% CIs. Risk ratios (RRs) with 95% CIs were used to evaluate the outcomes of ORRs. And the outcome of odds ratios (ORs) were used to assess grade ≥ 3 toxicities. The random-effect model (DerSimonian-Laird method) was used to calculate the pooled HR, RR and OR in direct and indirect meta-analysis. HRs were calculated by inverse-variance approach. ORs and RRs were calculated by Mantel–Haenszel method. The indirect comparison was based on the assumption of transitivity [12, 13]. When direct evidence was lacked between intervention A and intervention B, we could obtain indirect comparisons via common intervention C which associated intervention A with intervention B [14]. All calculations were conducted using STATA software [15–17], version 13.1 (StataCorp, College Station, TX, USA) with mvmeta, network, metareg, metan packages (http://fmwww.bc.edu/RePEc/bocode/m).

Node-spilt method was used to assess inconsistency [18]. Global or local heterogeneity among studies were evaluated by calculating *p* value, which was considered significant if p value was less than 0.05 [19]. Loop inconsistency was estimated by calculating IFs with 95% CIs, which was considered insignificant if 95% CI included 0. The possible bias of publication was detected by Egger’s test [20], which was considered significant if p value was less than 0.05.

## Results

### Study search

For initial literature search, a total of 4707 related studies were identified from the available databases. The detailed flow diagram of study selection process is shown in Fig. 1. We excluded 2223 studies due to duplication. Upon title and abstract screening, 212 studies remained. Only 22 studies were eligible after full-text screening and were included to conduct a final meta-analysis [21–42].

The detailed description of included studies is presented in Table S1. Four kinds of treatment were analyzed in order to compare the efficacy and safety of modified FOLFIRINOX and GEM-NAB. The number of patients with different treatments ranged from 12 to 632. No overlapping population existed in our analysis.

The characteristics of patients in the studies included in this study were showed in Table 1. The 22 studies involved a total of 7425 patients, with an average median age of 65.1 (range: 25–87) years. The majority of the patients was male (57.2%), with an ECOG score of 0–1 (88.5%), and with metastatic diseases (90.0%). 45.0% of the tumors occurred in head or neck of pancreas, and 48.4% occurred in body or tail of pancreas. With regard to the detailed sites of metastasis, 56.8% of the patients were with liver metastasis, 15.9% were with lymph-node metastasis, 17.8% were with peritoneal metastasis, and 13.9% were with lung metastasis. A total of 6351 patients were treated with the target therapies of our study and were used for further analysis, among which FOLFIRINOX accounted for 2659 cases (41.9%), GEM-NAB accounted for 1929 cases (30.4%), GEM accounted for 1420 cases (22.4%), and modified FOLFIRINOX accounted for 343 cases (5.4%) (Table 1 and Table S1).

**Table 1:** Characteristics of patients included in this study

Characteristics	No. of patients/No. of all patients	Percentage
No. of patients	7425/7425	100.0%
Average Median Age (range)	65.1 (25 to 87)	
Sex		
Male	3955/6912	57.2%
Female	2957/6912	42.8%
ECOG score		
0–1	4496/5083	88.5%
2+	587/5083	11.5%
Site		
Head or neck	1895/4209	45.0%
Body or tail	2037/4209	48.4%
Other*	277/4209	6.6%
Metastatic diseases	6684/7425	90.0%
Metastatic sites		
Liver	1825/3213	56.8%
Lymph nodes	511/3213	15.9%
Peritoneal	572/3213	17.8%
Lung	446/3213	13.9%
Other	401/3213	12.5%
Treatments		
FOLFIRINOX	2659/6351	41.9%
GEM-NAB	1929/6351	30.4%
GEM	1420/6351	22.4%
mFOLFIRINOX	343/6351	5.4%

Abbreviations: No.: number; ECOG: Eastern Cooperative Oncology Group; GEM: gemcitabine; GEM-NAB: Gemcitabine plus nab-paclitaxel; FOLFIRINOX: the combination of 5-fluorouracil, oxaliplatin, and irinotecan; mFOLFIRINOX: at least one of the drugs was reduced and/or the removal of 5-FU bolus in FOLFIRINOX

^*^ Including the cancers involved multiple sites or those originating from unknown subsites of pancreas

### Network meta-analysis of OS

The forest plots of network meta-analysis of OS are demonstrated in Fig. S1. Heterogeneity was detected and significant inconsistency was not observed in our data. The comparisons for hazards ratios of OS between different treatments are shown in Table 2. The therapy of modified FOLFIRINOX had worse survival benefit than the therapy of GEM-NAB (HR: 1.13; 95% CI: 0.78–1.63), but the difference was not significant. The use of gemcitabine monotherapy exhibited worse treatment efficacy than FOLFIRINOX (HR: 2.19; 95% CI: 1.77–2.71), GEM-NAB (HR: 2.03; 95% CI: 1.63–2.54) and modified FOLFIRINOX (HR: 2.29; 95% CI: 1.55–3.40).

**Table 2:** Indirect comparison of overall survival

HR of OS (95% CI)			
GEM			
2.03 (1.63–2.54) ^*^	GEM-NAB		
2.19 (1.77–2.71) ^*^	1.08 (0.92–1.25)	FOLFIRINOX	
2.29 (1.55–3.40) ^*^	1.13 (0.78–1.63)	1.05 (0.75–1.47)	mFOLFIRINOX

Abbreviation: HR: hazard ratio; OS: overall survival; CI: confidence interval; GEM: gemcitabine; GEM-NAB: Gemcitabine plus nab-paclitaxel; FOLFIRINOX: the combination of 5-fluorouracil, oxaliplatin, and irinotecan; mFOLFIRINOX: at least one of the drugs was reduced and/or the removal of 5-FU bolus in FOLFIRINOX

Notes: Comparisons between treatments were read from left to right, and the estimate (HR) with 95% CI for a given comparison was read in the intersection of two treatments. The value of estimates higher than 1 indicated that column-defining treatment had better efficacy

* Denotes *p*-value < 0.05

### Network meta-analysis of PFS

The forest plots of network meta-analysis of PFS are demonstrated in Fig. S2. Heterogeneity was not statistically significant in our analysis. Table 3 demonstrates indirect comparisons of hazards ratios of PFS between treatments, which shows similar results with OS. The survival data on PFS showed significantly worse efficacy in the treatment of modified FOLFIRINOX (HR: 1.19; 95% CI: 0.85–1.67) compared to GEM-NAB, but there was no significant difference. Gemcitabine monotherapy had worse treatments effects than modified FOLFIRINOX (HR: 3.22; 95% CI: 1.95–5.30), GEM-NAB (HR: 2.70; 95% CI: 1.79–4.08) and FOLFIRINOX (HR: 3.21; 95% CI: 2.10–4.89).

**Table 3:** Indirect comparison of progression free survival

HR of PFS (95% CI)			
GEM			
2.70 (1.79–4.08) ^*^	GEM-NAB		
3.21 (2.10–4.89) ^*^	1.19 (0.93–1.51)	FOLFIRINOX	
3.22 (1.95–5.30) ^*^	1.19 (0.85–1.67)	1.00 (0.78–1.29)	mFOLFIRINOX

Abbreviation: HR: hazard ratio; PFS: progression-free survival; CI: confidence interval; GEM: gemcitabine; GEM-NAB: Gemcitabine plus nab-paclitaxel; FOLFIRINOX: the combination of 5-fluorouracil, oxaliplatin, and irinotecan; mFOLFIRINOX: at least one of the drugs was reduced and/or the removal of 5-FU bolus in FOLFIRINOX

Notes: Comparisons between treatments were read from left to right, and the estimate (HR) with 95% CI for a given comparison was read in the intersection of two treatments. The value of estimates higher than 1 indicated that column-defining treatment had better efficacy

* Denotes *p*-value < 0.05

### Network meta-analysis of ORR

The forest plots of network meta-analysis of ORR are demonstrated in Fig. S3. Heterogeneity was not statistically significant in our analysis. The comparisons for risk ratios of ORR between different treatments are shown in Table 4, which were different from the results of OS or PFS. A better ORR was observed in GEM-NAB (RR: 1.43; 95% CI: 1.04–1.96) than modified FOLFIRINOX. Gemcitabine monotherapy showed less efficacy than other treatments.

**Table 4:** Indirect comparison of objective response rate

RR of ORR (95% CI)			
GEM			
0.21 (0.11–0.40) *	GEM-NAB		
0.25 (0.13–0.49) *	1.22 (0.95–1.58)	FOLFIRINOX	
0.30 (0.15–0.60) *	1.43 (1.04–1.96) *	1.17 (0.87–1.56)	mFOLFIRINOX

Abbreviation: HR: hazard ratio; ORR: objective response rate; CI: confidence interval; GEM: gemcitabine; GEM-NAB: Gemcitabine plus nab-paclitaxel; FOLFIRINOX: the combination of 5-fluorouracil, oxaliplatin, and irinotecan; mFOLFIRINOX: at least one of the drugs was reduced and/or the removal of 5-FU bolus in FOLFIRINOX

Notes: Comparisons between treatments were read from left to right, and the estimate (risk ratio, RR) with 95% confidence interval for a given comparison was read in the intersection of two treatments. The value of estimates higher than 1 indicated that column-defining treatment had better efficacy

* Denotes *p*-value < 0.05

### Toxicity

**Fig. 2:**
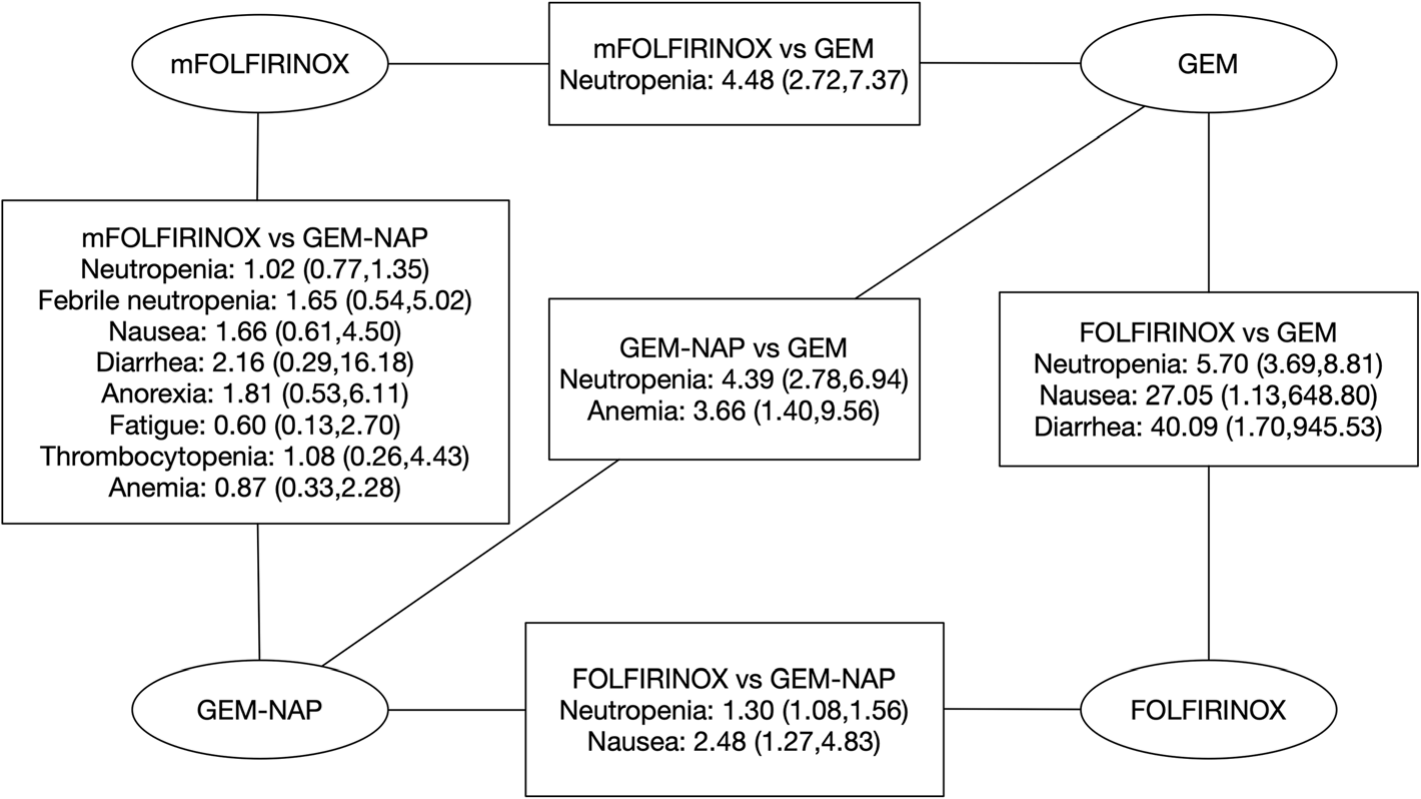
Indirect comparisons of toxicities: modified FOLFIRINOX vs. GEM-NAB. Data presented as odds ratio (OR) with 95% confidence interval (CI); the 95% confidence interval that did not contain the value of 1 represents as statistical significance. Statistically significant comparisons of toxicities between other treatments were also demonstrated

We demonstrated direct and indirect toxicity comparisons between different treatments and listed results in Fig. 2. Heterogeneity was not statistically significant in our analysis (*p* < 0.05). Patients treated with GEM-NAB or modified FOLFIRINOX showed similar risk of adverse events. Increasing risk of neutropenia, febrile neutropenia, thrombocytopenia, anorexia, nausea and diarrhea were observed in patients treated with modified FOLFIRINOX. And patients who received GEM-NAB showed a little higher risk of fatigue and anemia. But these results were not statistically significant. A higher risk of neutropenia was observed in modified FOLFIRINOX (OR: 4.48; 95% CI: 2.72–7.37), FOLFIRINOX (OR: 5.70; 95% CI: 3.69–8.81) and GEM-NAB (OR: 4.39; 95% CI: 2.78–6.94) than gemcitabine monotherapy. Gastrointestinal disorders such as nausea (OR: 27.05; 95% CI: 1.13–648.80) and diarrhea (OR: 40.09; 95% CI: 1.70–945.53) were more frequently observed in patients treated with FOLFIRINOX than gemcitabine monotherapy.

### Publication Bias

Publication bias of OS data was assessed by Egger’s test (*p* = 0.918). No publication bias was found in our analysis. And results were similar for PFS (Egger’s, *p* = 0.167) and ORR (Egger’s, *p* = 0.267). We further evaluated the potential sources of the heterogeneity by calculating *p* values. The p values were 0.512, 0.164 and 0.379 for OS, PFS and ORR, respectively, which showed no heterogeneity was found in our study.

## Discussion

Modified FOLFIRINOX and GEM-NAB are frequently used first-line treatments for advanced PC. Previous studies have conducted efficacy comparisons between modified FOLFIRINOX and FOLFIRINOX [8] or between GEM-NAB and FOLFIRINOX [43], and the outcomes were similar in these comparisons. However, until now, relative effects of modified FOLFIRINOX and GEM-NAB treatments are unknown as no direct comparisons have been conducted. This study performed a network meta-analysis of four kinds of treatments, including modified FOLFIRINOX, FOLFIRINOX, GEM-NAB and gemcitabine monotherapy, on survival outcomes and adverse effects. Thus, our analysis is unique of its kind and could provide some guidance to treatment selection in the first-line setting for advanced PC.

Chemotherapy is the cornerstone of advanced PC due to its invasive biological characteristics. It is well known that FOLFIRINOX is one of the standard treatments for advanced PC. Oxaliplatin, irinotecan and 5-FU were reported to show synergistic antitumor effects in several studies especially in metastatic PCs [44–46]. In a Phase III clinical trial [47], gemcitabine was associated with an increase in survival of 5.65 months compared with that of 4.41 months in 5-FU. Gemcitabine combined with nab-paclitaxel could lengthen the median OS to 8.5 months compared with 6.7 months in the gemcitabine monotherapy group. In 2013, based on the results of the MPACT trial [4], the GEM-NAB combination was approved for advanced PC in the first-line setting. When compared to gemcitabine alone indirectly, the mOS among patients treated with FOLFIRINOX or GEM-NAB were 11.1 months and 8.5 months, mPFS were 6.4 and 5.5 months, and ORRs were 31.6 and 23% respectively in ACCORD and MPACT pivotal studies [4, 9]. Thus, there were more therapeutic benefits from FOLFIRINOX than GEM-NAB in this cohort. However, previous meta-analysis based on 16 retrospective studies from Italy reported that the survival outcomes were similar between FOLFIRINOX and GEM-NAB [43], with HR for PFS of 0.88 (95% CI 0.71–1.1, *p* = 0.26) and HR for OS of 0.99 (95% CI 0.84–1.16, *p* = 0.9). In our meta-analysis, the treatment efficacy was also similar between FOLFIRINOX and GEM-NAB, which was same with previous studies.

FOLFOXIRI, a dosage-modified version of FOLFIRINOX, has been widely utilized and has shown satisfying outcomes in metastatic colorectal cancer [48]. An observational study of 137 patients from Italy reported that median OS and PFS were 8.0 months and 12.0 months respectively with metastatic PC treated with modified FOLFIRINOX (FOLFOXIRI) [49]. In view of all related retrospective studies of modified FOLFIRINOX and GEM-NAB, our indirect meta-analysis showed that survival outcomes were similar in these two treatments. The results of OS and PFS showed better treatment efficacy in modified FOLFIRINOX group than GEM-NAB group. But this difference was not statistically significant. As for results of ORR, GEM-NAB demonstrated more benefits than modified FOLFIRINOX, and this difference was statistically significant. Response rate reflects the short-term efficiency of the chemotherapeutic agents, thus the results showed that gemcitabine plus nab-paclitaxel has higher therapeutic efficiency. However, the similar survival between these two treatments reflected that the efficiency of GEM-NAB had not been transferred into benefits in the survival in the clinical practice. In the treatments of patients with PC, there are many factors which may influence the outcome of patients, including socioeconomic factors, second- or third-line treatments, follow up, patient compliance et al.

Treatment-related adverse effects were much severe in patients who received FOLFIRINOX (grade ≥ 3: neutropenia 45.7%, fatigue 23.6%, and diarrhea 12.7% of patients) than that of GEM-NAB (grade ≥ 3: neutropenia 38%, fatigue 7%, and diarrhea 6% of patients) in the PRODIGE4/ACCORD11 trial [4, 9, 50], which are the major concerns to impede the wide application of FOLFIRINOX. Thus, dose reduction of FOLFIRINOX have been conducted in several groups to reduce FOLFIRINOX-related toxicities. In a UK retrospective research [51], a lower rate of neutropenia was reported after dosage reduction of irinotecan and omission of 5-FU bolus than that in PRODIGE4/ACCORD11 trial. Dosage reduction of irinotecan and 5-FU bolus were also reported to decrease the risk of neutropenia and vomiting in a US phase II trial [52]. A retrospective research from South Korea found that the toxicity of modified FOLFIRINOX was less severe compared to FOLFIRINOX [26]. A previous meta-analysis from China found that modified FOLFIRINOX could reduce toxicity without compromising treatment efficacy compared to standard FOLFIRINOX [8]. However, the benefits from modified FOLFIRINOX were based only on comparisons with standard FOLFIRINOX. And the choice of modified FOLFIRINOX or GEM-NAB has been controversial due to the lack of evidence comparing the toxicity between two treatments. For grade ≥ 3 adverse events, our analysis showed that the risk was higher in patients treated with modified FOLFIRINOX than GEM-NAB including neutropenia, febrile neutropenia, thrombocytopenia, anorexia, nausea and diarrhea. And lower risk of fatigue and anemia was observed in the modified FOLFIRINOX group than that of the GEM-NAB group. However, our results did not significantly differ. Our analysis showed a similar toxicity profile in advanced pancreatic cancer patients treated with modified FOLFIRINOX compared to GEM-NAB, and provided some guidance to medical participants in treatment selection.

Many factors should be considered when formulating optimal treatment for PC. Relapse-free survival duration, for example, is an important factor in the decision between FOLFIRINOX and GEM-NAB for the relapse PCs. Generally, GEM-NAB is mainly used in the case of patient relapse occurring later than 6 months. In some cases, GEM-NAB is also usable in patients who relapse occurred for up to 6 months or less, such as those relapsing after completion of adjuvant chemotherapy including FOLFIRINOX or S-1. When the relapse occurring within 6 months, FOLFIRINOX is the main therapies if the patients have preferable health conditions, as the triplet therapy might has a worse toxicity profile compared to GEM-NAB [43]. Furthermore, patients should be notified of the costs of relevant supportive care such as apply of anti-emetics or pegfilgrastim [53]. Costs are not only based on chemotherapy drug itself but also on supportive treatment determined by severity and frequency of adverse effects. Previous study found that FOLFIRINOX therapy was more expensive than GEM-NAB [54]. Treatment patterns and economic outcomes between modified FOLFIRINOX and GEM-NAB were still unknown. However, modified FOLFIRINOX could reduce the therapy-related toxicity and costs compared with FOLFIRINOX, and has potential to use less or similar costs as that of GEM-NAB to archive similar therapy effects. The newest phase II clinical trial supported our results, with a two-year OS of 47% in modified FOLFIRINOX and 48% in GEM-NAB, which showed no prognosis differences [55]. We expected more studies to demonstrate whether modified FOLFIRINOX could be equal to or superior than GEM-NAB.

The latest clinical data were utilized in order to conduct indirect comparisons between different drug combinations. But this study has some clear limitations. Firstly, the results of analysis might be overestimated due to bias of small sample size. Further trials were required to specify the estimation of patients benefited. Secondly, our analysis was based on retrospective studies. Thus, patients were not randomly selected or distributed, and factors including patients age, performance status, tumor burden or disease stage were not adequately evaluated which might cause arms unbalanced. Thirdly, the data in terms of patients’ baseline characteristics and follow up were not complete. The evaluation of PFS or ORR were subjective to a certain extent with obvious bias in which the heterogeneity could not be avoided. Despite several limitations of this network meta-analysis, it is the initial work comparing efficacy and safety indirectly between modified FOLFIRINOX and GEM-NAB among PC patients.

## Conclusions

The current evidence showed that though GEM-NAB had higher ORR than modified FOLFIRINOX in the indirect analysis, the survival and toxicity of these two therapies were similar. Many factors should be considered for in the formulation of optimal treatment, and our meta-analysis could provide some guidance to treatment selection in the first-line setting for advanced PC. The use of both modified FOLFIRINOX and GEM-NAB as first-line therapy in patients with advanced PC should be promising in the future researches.

## Supplementary Information

**Additional file 1.** Supplementary tables and figures.

**Additional file 2.** Detailed search strategy.

**Additional file 3.** PRISMA NMA checklist of items to include when reporting a systematic review involving a network meta-analysis

## Data Availability

All data generated or analyzed during this study are included in this published article and referenced articles are listed in the References section.

## Abbreviations

GEM: gemcitabine
GEM-NAB: gemcitabine plus nab-paclitaxel
mFOLFIRINOX: modified FOLFIRINOX
PC: pancreatic cancer
OS: overall survival
PFS: progression-free survival
ORR: objective response rate
5-FU: 5-fluorouracil
CR: complete response
PR: partial response
SD: stable disease
PD: progressive disease
HRs: hazard ratios
RRs: risk ratios
ORs: odds ratios
CIs: confidence intervals
