# Supplementary material for: Meta-analysis and indirect treatment comparison of modified FOLFIRINOX and gemcitabine plus nab-paclitaxel as first-line chemotherapy in advanced pancreatic cancer
Source: BMC Cancer. 2021 Jul 23;21:853. doi: 10.1186/s12885-021-08605-x (PMC8306351; doi:10.1186/s12885-021-08605-x)
Supplement: Supplementary file 1 — Additional file 1. Supplementary tables and figures. [file 12885_2021_8605_MOESM1_ESM.docx]

**Supplementary table and figure legends**

**Table S1.** Characteristics of included studies.

**Figure S1.** Forest plots for the comparison of overall survival (OS) for different types of treatments. Hazard ratios (HRs) and 95% confidence interval (CIs) indicate the relative efficacy under the corresponding endpoint.

Notes: GEM: gemcitabine; GEM-NAB: Gemcitabine plus nab-paclitaxel; FOLFIRINOX: the combination of 5-fluorouracil, oxaliplatin, and irinotecan; mFOLFIRINOX: at least one of the drugs was reduced and/or the removal of 5-FU bolus in FOLFIRINOX.

**Figure S2.** Forest plots for the comparison of progression-free survival (PFS) for different types of treatments. Hazard ratios (HRs) and 95% confidence interval (CIs) indicate the relative efficacy under the corresponding endpoint.

Notes: GEM: gemcitabine; GEM-NAB: Gemcitabine plus nab-paclitaxel; FOLFIRINOX: the combination of 5-fluorouracil, oxaliplatin, and irinotecan; mFOLFIRINOX: at least one of the drugs was reduced and/or the removal of 5-FU bolus in FOLFIRINOX.

**Figure S3.** Forest plots for the comparison of objective response rate (ORR) for different types of treatments. Rate ratios (HRs) and 95% confidence interval (CIs) indicate the relative efficacy under the corresponding endpoint.

Notes: GEM: gemcitabine; GEM-NAB: Gemcitabine plus nab-paclitaxel; FOLFIRINOX: the combination of 5-fluorouracil, oxaliplatin, and irinotecan; mFOLFIRINOX: at least one of the drugs was reduced and/or the removal of 5-FU bolus in FOLFIRINOX.

**Table S1**. Characteristics of included studies.

| Author | Year of publication | Country | Start Time | End Time | PS | Number of patients | Median age | Males (%) | median follow-up | treatment |
| --- | --- | --- | --- | --- | --- | --- | --- | --- | --- | --- |
| Ohba, A. | 2018 | Japan | 2014 | 2016 | NA | 26 | NA | NA | NA | mFOLFIRINOX |
|  |  |  |  |  |  | 30 | NA | NA |  | FOLFIRINOX |
| Watanabe, K. | 2017 | Japan | 2013 | 2015 | 0-1 | 70 | NA | 66% | 27.5 months | mFOLFIRINOX |
|  |  |  |  |  |  | 65 | NA | 69% | 22.1 months | GEM-NAB |
| Kang, H. | 2018 | South Korea | 2013.1 | 2017.7 | 0-1 | 42 | 57 | 69.3% | 11.1 months | mFOLFIRINOX |
|  |  |  |  |  |  | 88 | 63.5 | 42.9% | 10.3 months | FOLFIRINOX |
| Vienot, A. | 2020 | France | 2011.1 | 2017.12 | 0-2 | 165 | 62.5 | 58.8% | 61.4 months (43.2-87.9) | mFOLFIRINOX |
|  |  |  |  |  |  | 124 | 60.2 | 58.9% | 30.8 months (23.0-NA) | FOLFIRINOX |
| de Jesus, V.H.F | 2018 | Brazil | 2010.1 | 2016.12 | 0-2 | 40 | 62.0 | 50.0% | 28.6 months (20.8–NA) | mFOLFIRINOX |
|  |  |  |  |  |  | 60 | 56.0 | 55.0% |  | FOLFIRINOX |
| Nakazawa, J. | 2019 | Japan | 2013.12 | 2018.6 | 0-2 | 92 | 68 | NA | 14.9 months (0.7-30.9) | GEM-NAB |
|  |  |  |  |  |  | 119 | 61 | NA | 13.1 months (1.3-40.8) | FOLFIRINOX |
| Wang, Y. | 2017 | Canada | 2014.8 | 2016.1 | 0-2 | 87 | 68.3 | 58.6% | 8.5 months | GEM-NAB |
|  |  |  |  |  |  | 92 | 60.35 | 47.8% |  | FOLFIRINOX |
|  |  |  |  |  |  | 46 | 74.3 | 60.9% |  | GEM |
| Kim, S | 2018 | USA | 2015.4 | 2015.12 | 0-4 | 337 | 64.59 | 64.7% | 10.7 months | GEM-NAB |
|  |  |  |  |  |  | 317 | 59.03 | 67.2% |  | FOLFIRINOX |
| Muranaka, T. | 2017 | Japan | 2013.12 | 2015.9 | 0-1 | 22 | 66.5 | 54.5% | 8.3 months | GEM-NAB |
|  |  |  |  |  |  | 16 | 63 | 62.5% | 11.9 months | FOLFIRINOX |
| Papneja, N. | 2019 | Canada | 2011 | 2016 | 0-3 | 33 | 64 | 52% | 8 months | GEM-NAB |
|  |  |  |  |  |  | 86 | 59 | 62% |  | FOLFIRINOX |
| Cho, I | 2018 | Korea | 2015 | NA | NA | 81 | 54 | NA | 7.9 months (1.5-23.4) | GEM-NAB |
|  |  |  |  |  |  | 86 | 65 | NA |  | FOLFIRINOX |
| Williet, N. | 2019 | France | 2015.6 | 2018.6 | 0-2 | 109 | 68.1 | 49.5% | NA | GEM-NAB |
|  |  |  |  |  |  | 107 | 61.8 | 59.8% |  | FOLFIRINOX |
| Tahara, J. | 2018 | Japan | 2014.3 | 2017.4 | 0-1 | 15 | 63 | 53.3% | NA | GEM-NAB |
|  |  |  |  |  |  | 12 | 62 | 58.3% |  | FOLFIRINOX |
| Javed, M.A. | 2018 | Sweden | 2012 | 2015 | 0-3 | 81 | 63.9 | 55.6% | NA | GEM-NAB |
|  |  |  |  |  |  | 204 | 60.0 | 58.8% |  | FOLFIRINOX |
|  |  |  |  |  |  | 450 | 69.0 | 47.6% |  | GEM |
| Latenstein, A.E.J | 2020 | Netherlands | 2007 | 2016 | 0-4 | 70 | 68 | 50.0% | NA | GEM-NAB |
|  |  |  |  |  |  | 436 | 61 | 56.2% |  | FOLFIRINOX |
|  |  |  |  |  |  | 182 | 70 | 54.4% |  | GEM |
| Chan, K.K.W | 2019 | Canada | 2015.4 | 2017.3 | NA | 498 | 69.14 | 60.2% | NA | GEM-NAB |
|  |  |  |  |  |  | 632 | 61.83 | 54.6% |  | FOLFIRINOX |
| Terashima, T. | 2018 | Japan | 2009.1 | 2015.7 | 0-4 | 20 | NA | NA | 5.6 months (0.03-42.4) | GEM-NAB |
|  |  |  |  |  |  | 47 | NA | NA |  | FOLFIRINOX |
|  |  |  |  |  |  | 365 | NA | NA |  | GEM |
| Kang, J | 2018 | South Korea | 2013.1 | 2016.12 | 0-2 | 122 | 60 | 68.9% | NA | GEM-NAB |
|  |  |  |  |  |  | 149 | 62 | 71.8% |  | FOLFIRINOX |
| Barrera, I. | 2019 | Canada | 2010 | 2018 | NA | 41 | NA | NA | NA | GEM-NAB |
|  |  |  |  |  |  | 60 | NA | NA |  | FOLFIRINOX |
|  |  |  |  |  |  | 60 | NA | NA |  | GEM |
| Kasi, A. | 2017 | USA | 2011.1 | 2016.11 | 0-1 | 47 | 63 | 54% | NA | GEM-NAB |
|  |  |  |  |  |  | 107 | 61 | 57% |  | FOLFIRINOX |
| Lee, J.-C | 2019 | Korean | 2011 | 2017 | NA | 182 | 68 | 39% | NA | GEM-NAB |
|  |  |  |  |  |  | 231 | 60 | 51% |  | FOLFIRINOX |
| Longo Muñoz, F. | 2019 | Spain | 2011.1 | 2018.5 | NA | NA | NA | NA | NA | GEM-NAB |
|  |  |  |  |  |  | NA | NA | NA |  | FOLFIRINOX |

PS: ECOG performance status. GEM: gemcitabine; GEM-NAB: Gemcitabine plus nab-paclitaxel; FOLFIRINOX: the combination of 5-fluorouracil, oxaliplatin, and irinotecan; mFOLFIRINOX: at least one of the drugs was reduced and/or the removal of 5-FU bolus in FOLFIRINOX.


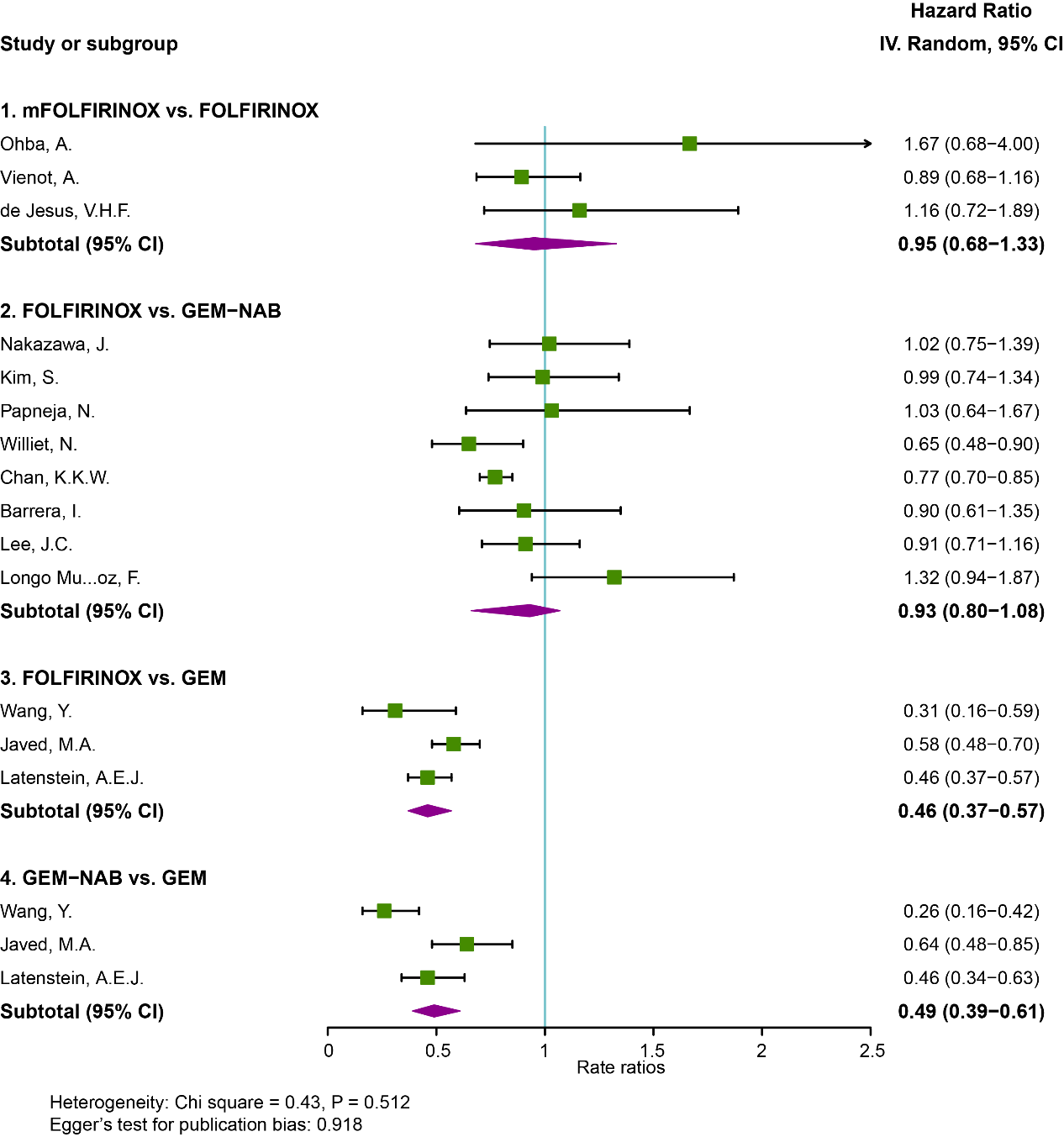


**Figure S1.** Forest plots for the comparison of overall survival (OS) for different types of treatments. Hazard ratios (HRs) and 95% confidence interval (CIs) indicate the relative efficacy under the corresponding endpoint.

Notes: GEM: gemcitabine; GEM-NAB: Gemcitabine plus nab-paclitaxel; FOLFIRINOX: the combination of 5-fluorouracil, oxaliplatin, and irinotecan; mFOLFIRINOX: at least one of the drugs was reduced and/or the removal of 5-FU bolus in FOLFIRINOX.


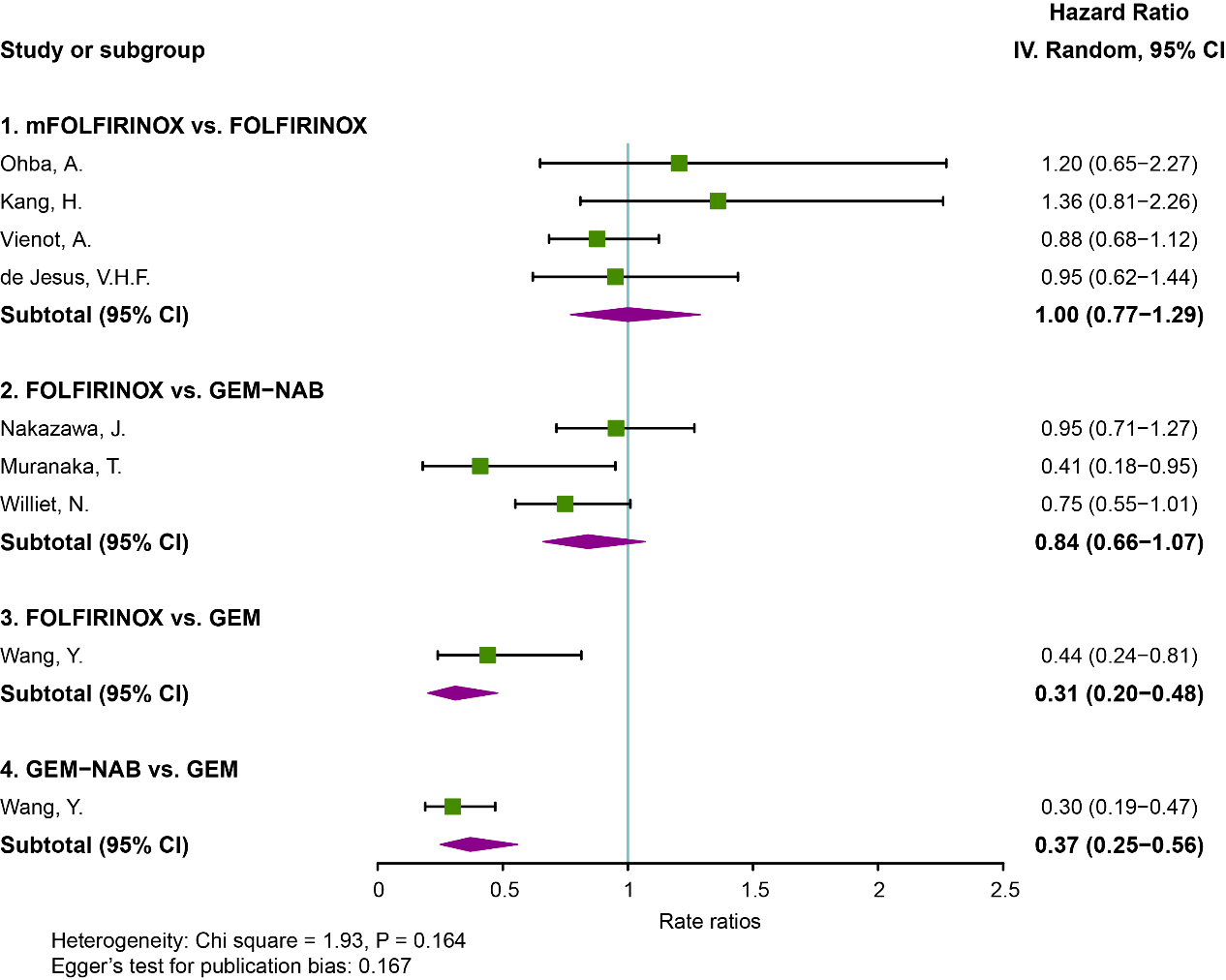


**Figure S2.** Forest plots for the comparison of progression-free survival (PFS) for different types of treatments. Hazard ratios (HRs) and 95% confidence interval (CIs) indicate the relative efficacy under the corresponding endpoint.

Notes: GEM: gemcitabine; GEM-NAB: Gemcitabine plus nab-paclitaxel; FOLFIRINOX: the combination of 5-fluorouracil, oxaliplatin, and irinotecan; mFOLFIRINOX: at least one of the drugs was reduced and/or the removal of 5-FU bolus in FOLFIRINOX.


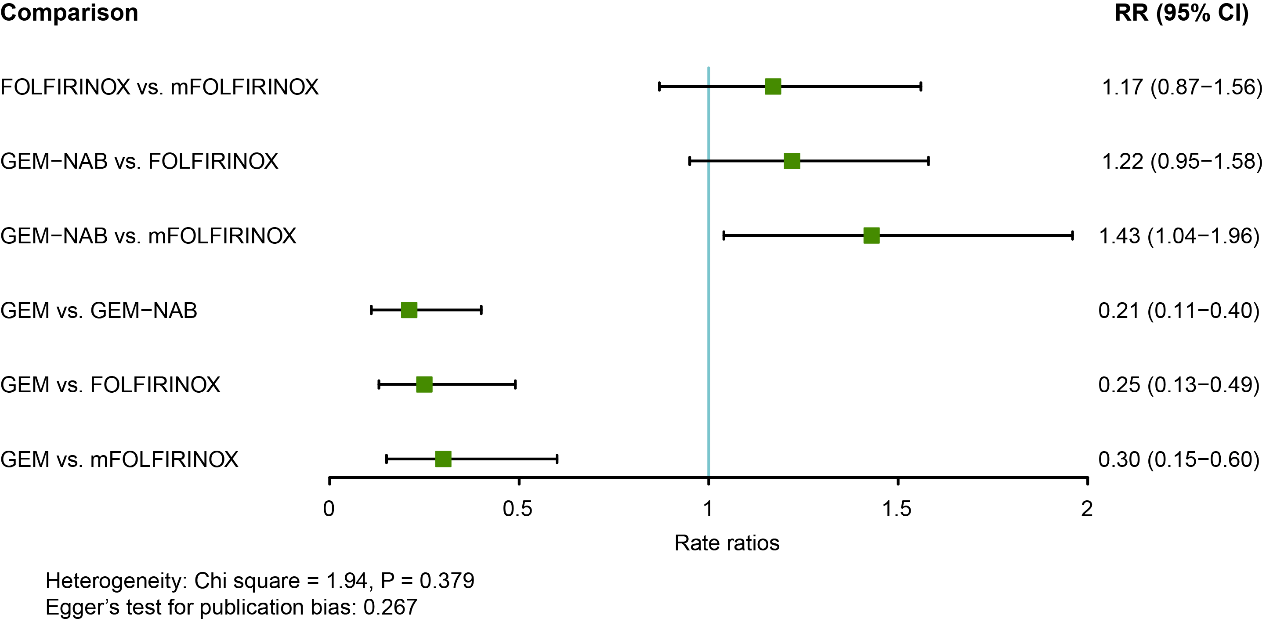


**Figure S3.** Forest plots for the comparison of objective response rate (ORR) for different types of treatments. Rate ratios (HRs) and 95% confidence interval (CIs) indicate the relative efficacy under the corresponding endpoint.

Notes: GEM: gemcitabine; GEM-NAB: Gemcitabine plus nab-paclitaxel; FOLFIRINOX: the combination of 5-fluorouracil, oxaliplatin, and irinotecan; mFOLFIRINOX: at least one of the drugs was reduced and/or the removal of 5-FU bolus in FOLFIRINOX.
