# Supplementary material for: Meta-analysis and indirect treatment comparison of modified FOLFIRINOX and gemcitabine plus nab-paclitaxel as first-line chemotherapy in advanced pancreatic cancer
Source: BMC Cancer. 2021 Jul 23;21:853. doi: 10.1186/s12885-021-08605-x (PMC8306351; doi:10.1186/s12885-021-08605-x)
Supplement: Supplementary file 2 — Additional file 2. Detailed search strategy. [file 12885_2021_8605_MOESM2_ESM.docx]

Supplementary appendix for: **Meta-analysis and indirect treatment comparison of modified FOLFIRINOX and gemcitabine plus nab-paclitaxel as first-line chemotherapy in advanced pancreatic cancer**

**Detailed search strategy**

**Pubmed**

#1 ("Pancreatic Neoplasms"[Mesh]) OR (Neoplasm, Pancreatic) OR (Pancreatic Neoplasm) OR (Pancreas Neoplasms) OR (Neoplasm, Pancreas) OR (Neoplasms, Pancreas) OR (Pancreas Neoplasm) OR (Neoplasms, Pancreatic) OR (Cancer of Pancreas) OR (Pancreas Cancers) OR (Pancreas Cancer) OR (Cancer, Pancreas) OR (Cancers, Pancreas) OR (Pancreatic Cancer) OR (Cancer, Pancreatic) OR (Cancers, Pancreatic) OR (Pancreatic Cancers) OR (Cancer of the Pancreas)

#2 ("Fluorouracil"[Mesh]) OR (5-FU) OR (Fluoruracil) OR (5-Fluorouracil) OR (5 Fluorouracil) OR (5FU) OR (5-FU Lederle) OR (5 FU Lederle) OR (Riemser Brand of Fluorouracil) OR (5-FU medac) OR (5 FU medac) OR (medac Brand of Fluorouracil) OR (5-HU Hexal) OR (5 HU Hexal) OR (Hexal Brand of Fluorouracil) OR (Adrucil) OR (Teva Brand of Fluorouracil) OR (Fluorouracil Teva Brand) OR (Carac) OR (Dermik Brand of Fluorouracil) OR (Efudix) OR (Dermatech Brand of Fluorouracil) OR (Roche Brand of Fluorouracil) OR (Fluoro-Uracile ICN) OR (Fluoro Uracile ICN) OR (ICN Brand of Fluorouracil) OR (CSP Brand of Fluorouracil) OR (Efudex) OR (Fluoroplex) OR (Flurodex) OR (Allergan Brand of Fluorouracil) OR (Fluorouracil Mononitrate) OR (Fluorouracil Monopotassium Salt) OR (Fluorouracil Monosodium Salt) OR (Fluorouracil Potassium Salt) OR (Fluorouracil-GRY) OR (Fluorouracil GRY) OR (Gry Brand of Fluorouracil) OR (Fluorouracile Dakota) OR (Dakota, Fluorouracile) OR (Dakota Brand of Fluorouracil) OR (Fluorouracilo Ferrer Far) OR (Ferrer Brand of Fluorouracil) OR (Fluracedyl) OR (Pharmachemie Brand of Fluorouracil Monosodium Salt) OR (Haemato-fu) OR (Haemato fu) OR (Haemato Brand of Fluorouracil) OR (Neofluor) OR (Neocorp Brand of Fluorouracil) OR (Onkofluor) OR (Onkoworks Brand of Fluorouracil) OR (Ribofluor) OR (ribosepharm Brand of Fluorouracil) OR (5-Fluorouracil-biosyn) OR (5 Fluorouracil biosyn) OR (biosyn Brand of Fluorouracil)

#3 ("oxaliplatin" [Supplementary Concept]) OR (1,2-diamminocyclohexane(trans-1)oxolatoplatinum(II)) (oxalato-(1,2-cyclohexanediamine)platinum II) OR (L-OHP cpd) OR (oxaliplatine) OR (1,2-diaminocyclohexane platinum oxalate) OR (platinum(II)-1,2-cyclohexanediamine oxalate) OR (cis-oxalato-(trans-l)-1,2-diaminocyclohexane-platinum(II)) OR (oxaliplatin, (SP-4-3-(cis))-isomer) OR (oxaliplatin, (SP-4-2-(1R-trans))-isomer) OR (oxaliplatin, (SP-4-2-(1S-trans))-isomer) OR (ACT 078) OR (ACT-078) OR (Eloxatine) OR (Sanofi Synthelabo brand of oxaliplatin) OR (Sanofi brand of oxaliplatin) OR (Eloxatin)

#4 ("Leucovorin"[Mesh]) OR (Leukovorum) OR (Leukovorin) OR (Folinic Acid) OR (Acid, Folinic) OR (Folinic Acid-SF) OR (Folinic Acid SF) OR (Leucovorin, (D)-Isomer) OR (Leucovorin, (DL)-Isomer) OR (Leucovorin, (R)-Isomer) OR (Leucovorin, Calcium (1:1) Salt) OR (Leucovorin, Calcium (1:1) Salt, Pentahydrate) OR (Leucovorin, Monosodium Salt) OR (Monosodium Salt Leucovorin) OR (N(5)-Formyltetrahydrofolate) OR (5-Formyltetrahydropteroylglutamate) OR (5 Formyltetrahydropteroylglutamate) OR (5-Formyltetrahydrofolate) OR (5 Formyltetrahydrofolate) OR (Wellcovorin) OR (Citrovorum Factor) OR (Factor, Citrovorum) OR (Calcium Leucovorin) OR (Leucovorin, Calcium) OR (Calcium Folinate) OR (Folinate, Calcium) OR (Leucovorin, Calcium (1:1) Salt, (DL)-Isomer)

#5 ("irinotecan" [Supplementary Concept]) OR (Irrinotecan) OR (Camptosar) OR (SN 38 11) OR (SN-38-11) OR (NK012 compound) OR (SN 38) OR (SN38 cpd) OR (SN-38) OR (7-ethyl-10-hydroxycamptothecin) OR (irinotecan hydrochloride) OR (camptothecin-11) OR (CPT 11) OR (CPT-11)

#6 FOLFIRINOX

#7 "gemcitabine" [Supplementary Concept] OR (dFdCyd) OR (gemcitabine hydrochloride) OR (LY 188011) OR (LY-188011)

#8 "Albumin-Bound Paclitaxel"[Mesh] OR (Albumin Bound Paclitaxel) OR (Paclitaxel, Albumin-Bound) OR (Protein-Bound Paclitaxel) OR (Paclitaxel, Protein-Bound) OR (Protein Bound Paclitaxel) OR (Abraxane) OR (ABI007) OR (ABI-007) OR (ABI 007) OR (nab-PTX)

#9 #2 and #3 and #4 and #5

#10 #6 or #9

#11 #7 and #8

#12 #10 or #11

#13 #12 and #1

**Cochrane**:

#1 (Pancreatic Neoplasms) or (Neoplasm, Pancreatic) or (Pancreatic Neoplasm) or (Pancreas Neoplasms) or (Neoplasm, Pancreas) or (Neoplasms, Pancreas) or (Pancreas Neoplasm) or (Neoplasms, Pancreatic) or (Cancer of Pancreas) or (Pancreas Cancers) or (Pancreas Cancer) or (Cancer, Pancreas) or (Cancers, Pancreas) or (Pancreatic Cancer) or (Cancer, Pancreatic) or (Cancers, Pancreatic) or (Pancreatic Cancers) or (Cancer of the Pancreas)

#2 (FOLFIRINOX)

#3 (Fluorouracil) or (5FU) or (Fluoruracil) or (5 Fluorouracil) or (5 FU Lederle) or (Riemser Brand of Fluorouracil) or (5 FU medac) or (medac Brand of Fluorouracil) or (5 HU Hexal) or (Hexal Brand of Fluorouracil) or (Adrucil) or (Teva Brand of Fluorouracil) or (Fluorouracil Teva Brand) or (Carac) or (Dermik Brand of Fluorouracil) or (Efudix) or (Dermatech Brand of Fluorouracil) or (Roche Brand of Fluorouracil) or (Fluoro Uracile ICN) or (ICN Brand of Fluorouracil) or (CSP Brand of Fluorouracil) or (Efudex) or (Fluoroplex) or (Flurodex) or (Allergan Brand of Fluorouracil) or (Fluorouracil Mononitrate) or (Fluorouracil Monopotassium Salt) or (Fluorouracil Monosodium Salt) or (Fluorouracil Potassium Salt) or (Fluorouracil GRY) or (Gry Brand of Fluorouracil) or (Fluorouracile Dakota) or (Dakota, Fluorouracile) or (Dakota Brand of Fluorouracil) or (Fluorouracilo Ferrer Far) or (Ferrer Brand of Fluorouracil) or (Fluracedyl) or (Pharmachemie Brand of Fluorouracil Monosodium Salt) or (Haemato fu) or (Haemato Brand of Fluorouracil) or (Neofluor) or (Neocorp Brand of Fluorouracil) or (Onkofluor) or (Onkoworks Brand of Fluorouracil) or (Ribofluor) or (ribosepharm Brand of Fluorouracil) or (5 Fluorouracil biosyn) or (biosyn Brand of Fluorouracil)

#4 (oxaliplatin) or (oxalate (1,2 cyclohexanediamine) platinum ii) or (l ohp cpd) or (1,2 diaminocyclohexane platinum oxalate) or (platinum (II) 1,2 cyclohexanediamine oxalate) or (bis oxalato (trans l) 1,2 diaminocyclohexane platinum (II)) or (oxaliplatin, (SP 4 3 (cis)) isomer) or (oxaliplatin, (SP 4 2 (1R trans)) isomer) or (oxaliplatin, (SP 4 2 (1S trans)) isomer) or (ACT 078) or (ACT 078) or (eloxatin) or (sanofi synthelabo brand of oxaliplatin) or (sanofi brand of oxaliplatin) or eloxatin

#5 (leucovorin) or (leukovorum) or (leukovorin) or (folinic acid) or (acid, folinic) or (folinic acid sf) or (folinic acid sf) or (leucovorin, (D) isomer) or (leucovorin, (DL) isomer) or (leucovorin, (R) isomer) or (leucovorin, calcium 1 1 salt) or (leucovorin, calcium 1 1 salt, pentahydrate) or (leucovorin, monosodium salt) or (monosodium salt leucovorin) or (N (5) formyltetrahydrofolate) or (5 Formyltetrahydropteroylglutamate) or (5 formyltetrahydropteroylglutamate) or (5 Formyltetrahydrofolate) or (5 formyltetrahydrofolate) or (wellcovorin) or (citrovorum factor) or (factor, citrovorum) or (calcium leucovorin) or (leucovorin, calcium) or (calcium folinate) or (folinate, calcium) or (leucovorin, calcium 1 1 salt, (DL) isomer)

#6 (irinotecan) or irinotecan or (camptosar) or (SN 38 11) or (SN 38 11) or (nk012 compound) or (SN 38) or (SN38 cpd) or (SN 38) or (7 ethyl 10 hydroxycamptothecin) or (irinotecan hydrochloride) or (camptothecin 11) or (CPT 11) or (CPT 11)

#7 (gemcitabine) OR (dFdCyd) OR (gemcitabine hydrochloride) OR (LY 188011) OR (LY-188011)

#8 (Albumin-Bound Paclitaxel) OR (Albumin Bound Paclitaxel) OR (Paclitaxel, Albumin-Bound) OR (Protein-Bound Paclitaxel) OR (Paclitaxel, Protein-Bound) OR (Protein Bound Paclitaxel) OR (Abraxane) OR (ABI007) OR (ABI-007) OR (ABI 007) OR (nab-PTX)

#9 #3 and #4 and #5 and #6

#10 #9 or #2

#11 #7 and #8

#12 #10 or #11

#13 #1 and #12

**EMBASE:**

((('folinic acid'/exp AND fluorouracil/exp AND irinotecan/exp AND oxaliplatin/exp AND 'drug combination'/exp) OR (Folfirinox):ab,ti) OR (gemcitabine/exp AND 'Albumin-Bound Paclitaxel'/exp 'drug combination'/exp) ) AND ('pancreas cancer'/de OR 'pancreas tumor'/de OR 'pancreas adenoma'/de OR 'pancreas adenocarcinoma'/de OR 'pancreas carcinoma'/de OR 'pancreas islet cell carcinoma'/de OR (pancrea* NEAR/3 (cancer* OR neoplas* OR tumo* OR adenocarcinom* OR carcinom* OR adenom*)):ab,ti)

**WEB OF SCIENCE:**

#1 TS= (Folfirinox)

#2 TS= (pancrea* AND (cancer* OR neoplas*OR tumo* OR adenocarcinom* OR carcinom* OR adenom*))

#3 TS= ((Fluor*uracil*) OR (5*FU) OR (5*HU Hexal) OR (Adrucil) OR (Carac) OR (Efudix) OR (Efudex) OR (Fluoroplex) OR (Flurodex) OR (Fluracedyl) OR (Haemato*fu) OR (Neofluor) OR (Onkofluor) OR (Ribofluor))

#4 TS= ((oxaliplatin) OR (L-OHP cpd) OR (oxaliplatine) OR (1,2-diaminocyclohexane platinum oxalate) OR (ACT*078) OR (Eloxatine) OR (Eloxatin))

#5 TS= ((Leu*ovorin) OR (Leukovorum) OR (Folinic Acid) OR (Wellcovorin) OR (Citrovorum Factor) OR (Calcium Folinate) OR (Formyltetrahydro*))

#6 TS= ((ir*inotecan) OR (Camptosar) OR (NK012 compound) OR (SN*38) OR (7-ethyl-10-hydroxycamptothecin) OR (camptothecin-11) OR (CPT*11))

#7 TS= ((gemcitabine) OR (dFdCyd) OR (gemcitabine hydrochloride) OR (LY 188011) OR (LY-188011))

#8 TS= ((Albumin-Bound Paclitaxel) OR (Albumin Bound Paclitaxel) OR (Paclitaxel, Albumin-Bound) OR (Protein-Bound Paclitaxel) OR (Paclitaxel, Protein-Bound) OR (Protein Bound Paclitaxel) OR (Abraxane) OR (ABI007) OR (ABI-007) OR (ABI 007) OR (nab-PTX))

#9 #6 AND #5 AND #4 AND #3

#10 #9 OR #1

#11 #7 AND #8

#12 #10 OR #11

#13 #12 AND #2
